# Apple-Derived Vesicles Orchestrate Bone Regeneration: *In Vitro* Proof of Concept

**DOI:** 10.3390/ijms27062719

**Published:** 2026-03-17

**Authors:** Giulia Brunello, Ilaria Vitali, Luna Ardondi, Maria Pia Cavaleri, Lucia Sileo, Marta Degasperi, Francesca Zalunardo, Kathrin Becker, Beryl Schwarz-Herzke, Stefano Sivolella, Luca Lovatti, Letizia Ferroni, Barbara Zavan

**Affiliations:** 1 Department of Oral Surgery, University Hospital of Düsseldorf, 40225 Düsseldorf, Germany; 2 Department of Orthodontics and Dentofacial Orthopedics, Charité—Universitätsmedizin Berlin, 14197 Berlin, Germany; 3 Department of Neurosciences, Dentistry Section, University of Padova, 35122 Padova, Italy; 4 Department of Medical Sciences, University of Ferrara, 44121 Ferrara, Italy; 5 Research and Development Melinda Institute, Cles, 38023 Trento, Italy; 6 Institute of Anatomy II, Medical Faculty, Heinrich Heine University, 40225 Düsseldorf, Germany; 7 Maria Cecilia Hospital, GVM Care and Research, 48033 Cotignola, Italy

**Keywords:** apple-derived extracellular vesicles, plant-derived extracellular vesicles, exosomes, bone regeneration, mesenchymal stem cells

## Abstract

The immune microenvironment critically influences bone healing, particularly in the oral cavity where inflammation and microbial biofilms can compromise regeneration. Plant-derived extracellular vesicles (PDEVs) offer a biocompatible means to modulate immune responses, and apple-derived extracellular vesicles (ADEVs) have shown antioxidant and anti-inflammatory activity, although their osteoregenerative potential remains unclear. Here, we investigate the indirect effects of ADEVs on bone regeneration by assessing how their immunomodulatory action on macrophages influences the osteogenic commitment of human dental pulp stem cells (DPSCs). ADEVs were isolated, characterized, and applied to THP-1-derived macrophages to evaluate polarization via morphology and immunofluorescence for M1 (iNOS) and M2 (ARG1) markers. Then, the extracellular vesicles (EVs) from untreated and ADEV-treated macrophages were isolated and applied to DPSCs. All EVs were efficiently internalized by both macrophages and DPSCs. Treated macrophages shifted toward an M2-like phenotype, and macrophage-derived EVs (MDEVs) promoted stem cell morphological features consistent with osteogenic activation. These findings suggest that ADEVs promote osteoregeneration indirectly by influencing macrophage polarization and modifying the osteoactive cargo of MDEVs, thereby supporting their potential in cell-free, immunomodulatory approaches for oral bone regeneration.

## 1. Introduction

The restoration of alveolar bone integrity remains one of the foremost priorities in oral and maxillofacial surgery, implantology, and periodontics [1,2,3]. Despite advances in surgical techniques and biomaterials, clinical outcomes in bone regeneration are often unpredictable, especially in patients with compromised healing capacity or chronic inflammation [4,5,6,7,8]. Traditional regenerative strategies largely focus on the delivery of osteoprogenitor cells, scaffolds, and growth factors [9,10,11,12,13]. However, emerging evidence underscores the pivotal role of the immune system, in particular of macrophage-mediated immune responses, in orchestrating the early phases of bone healing and determining long-term regenerative success [14,15,16,17].

Macrophages are dynamic cells of the innate immune system that exhibit plasticity and can polarize into distinct functional phenotypes in response to environmental stimuli. The pro-inflammatory M1 phenotype predominates in the initial stages of tissue injury, mediating pathogen clearance and cytokine release. In contrast, the M2 phenotype is associated with tissue remodeling, resolution of inflammation, and promotion of repair [18,19,20]. An effective regenerative process, especially in the context of oral tissues subjected to bacterial load and mechanical stress, requires a finely regulated temporal switch from the M1 to M2 phenotype. Dysregulation of this immunological balance can impair healing and contribute to fibrosis or chronic inflammation [21,22,23].

In recent years, extracellular vesicles (EVs) have emerged as promising tools for modulating intercellular communication and immune responses in regenerative contexts [24,25,26,27]. EVs are membrane-bound particles secreted by virtually all cell types and serve as carriers of signaling molecules, including proteins, RNAs, and lipids, that can influence target cell behavior [27,28]. Plant-derived extracellular vesicles (PDEVs) represent a novel class of EVs with unique advantages: they are naturally biocompatible, scalable, devoid of zoonotic risk, and capable of delivering bioactive molecules across biological barriers [29,30,31].

Among PDEVs, domestic apple (*Malus domestica*, Golden Delicious variety) has emerged as a highly accessible and biologically active source of nanovesicles with reproducible biochemical and functional properties. In our previous investigations, apple-derived extracellular vesicles (ADEVs) have been extensively isolated, dimensionally characterized (mean diameter ~115–150 nm), and quantified through tunable resistive pulse sensing (TRPS), nanoparticle tracking analysis (NTA), transmission electron microscopy (TEM), and protein content assessment, confirming morphological homogeneity and structural stability [24,25,26,27,28,29,30,31,32].

Comprehensive molecular profiling has demonstrated that ADEVs possess a complex and bioactive cargo. Transcriptomic and small RNA sequencing analyses revealed enrichment in regulatory miRNAs, including miR-146a, with demonstrated immunomodulatory potential. Proteomic characterization identified antioxidant enzymes (including SOD and peroxidase-related proteins), metabolic regulators, and stress-response proteins, while lipidomic analysis confirmed a membrane composition enriched in phosphatidylcholine, phosphatidylethanolamine, and glycolipid species that support vesicle stability and cellular uptake. Metabolomic profiling further indicated the presence of polyphenol-associated bioactive compounds consistent with the known antioxidant profile of *Malus domestica* [33,34].

Importantly, the biological safety of ADEVs has been rigorously evaluated. *In vitro* safety testing was performed according to internationally recognized OECD guidelines, including Ames test (OECD 471) for mutagenicity, reconstructed human corneal epithelium assay (OECD 492), skin corrosion (OECD 431), skin irritation (OECD 439), and sensitization assays (OECD 442C/442D KeratinoSens and h-CLAT models). Across all standardized assays, ADEVs demonstrated no genotoxic, cytotoxic, corrosive, or sensitizing effects, confirming a favorable toxicological profile. Cellular viability assays (MTT, LDH) in multiple mammalian cell lines further confirmed the absence of cytotoxicity even at biologically active concentrations [29].

Beyond *in vitro* evaluation, *in vivo* safety and efficacy have also been documented. Oral administration of ADEVs in a canine inflammatory bowel disease (IBD) model demonstrated not only clinical improvement and reduction in inflammatory markers (including calprotectin and secretory IgA normalization), but also restoration of intestinal mucosal architecture and modulation of gut microbiota composition. Specifically, ADEVs induced a significant shift toward eubiotic microbial populations, increasing beneficial genera (e.g., *Fusobacterium* and *Blautia*) while reducing opportunistic pathogens such as *Escherichia coli*. Ultrasound and endoscopic analyses confirmed improved mucosal integrity without evidence of adverse tissue reactions, supporting systemic biocompatibility and long-term safety [35].

Mechanistically, ADEVs exert a well-defined immunomodulatory action on macrophages. Our published data demonstrate that ADEVs are internalized by human macrophages and modulate their phenotype through a miR-146a–dependent pathway. This modulation results in downregulation of TLR4 signaling and inhibition of the NF-κB pathway, leading to decreased expression of pro-inflammatory mediators such as TNF-α, IL-6, IL-1β, and iNOS, alongside increased expression of anti-inflammatory and reparative markers including IL-10 and ARG1. This macrophage reprogramming toward a pro-resolving phenotype represents a central mode of action [29].

In dermal fibroblasts, transcriptomic analyses further revealed downregulation of canonical inflammatory pathways and decreased expression of Matrix Metalloproteases, accompanied by increased collagen type I and type III synthesis, confirming an extracellular matrix–preserving and regenerative profile. The integrated immunomodulatory and pro-regenerative signaling supports a coordinated multi-pathway mechanism rather than a single-target effect.

Taken together, ADEVs represent a biologically safe, molecularly characterized, and mechanistically supported plant-derived vesicle system capable of modulating oxidative stress, immune activation, extracellular matrix remodeling, and microbiome balance. However, despite the growing body of evidence supporting their antioxidant, anti-inflammatory, and regenerative potential, their specific role in orchestrating immune-driven bone regenerative processes, particularly through macrophage polarization and downstream effects on bone-forming cells, remains insufficiently explored and warrants further investigation. [36,37]. In light of our previously reported *in vitro* and *in vivo* data demonstrating the biological safety, immunomodulatory properties, and systemic tolerability of apple-derived extracellular vesicles (ADEVs), we propose the following working hypothesis. Our prior investigations have consistently shown that ADEVs interact with mammalian cells, are efficiently internalized, and modulate inflammatory signaling pathways without inducing cytotoxic, genotoxic, or sensitizing effects. These findings suggest that ADEVs are capable of influencing cellular behavior in a controlled and biologically compatible manner [35].

Building on this evidence, we hypothesize that ADEVs may exert upstream regulatory effects on human macrophages, not by forcing a rigid or binary polarization state, but by promoting a phenotypic modulation toward a more regulatory and pro-resolving functional profile. Rather than inducing a M1-to-M2 conversion, ADEV exposure may subtly reshape macrophage signaling dynamics, attenuating pro-inflammatory pathways while favoring characteristics commonly associated with reparative, M2-like macrophages. Such modulation may involve the regulation of canonical inflammatory mediators, including NF-κB-dependent pathways, alongside the upregulation of anti-inflammatory and tissue-supportive factors [29,30,31,32,33,34,36].

Within this framework, we further speculate that extracellular vesicles secreted by ADEV-conditioned macrophages may represent a secondary level of biological communication capable of influencing downstream cellular targets. In particular, we hypothesize that these macrophage-derived vesicles could interact with mesenchymal stem cells (MSCs), contributing to the regulation of osteogenic commitment. This effect may not be limited to differentiation alone, but could also involve modulation of extracellular matrix remodeling, metabolic activity, and paracrine signaling.

Moreover, considering that effective bone regeneration requires the coordinated interplay between immune regulation, stem cell activation, and vascular network formation, we propose that ADEVs may contribute to this complex regenerative cascade by acting on multiple interconnected pathways. In this context, macrophage-mediated modulation may influence MSC behavior not only by enhancing osteogenic differentiation markers but also by supporting angiogenic signaling and vascularization processes, which are essential for nutrient supply, cellular survival, and long-term tissue integration.

Therefore, rather than focusing on a single mechanistic axis, our hypothesis is that ADEVs participate in the orchestration of a multifactorial regenerative microenvironment. Through subtle immunomodulatory priming of macrophages and the subsequent release of functionally active vesicles, ADEVs may indirectly contribute to bone tissue regeneration by integrating immune resolution, osteogenic differentiation, and vascular support into a coordinated biological response (Figure 1).

This approach reconstructs a sequential vesicle-mediated communication pathway in which ADEVs modulate innate immune cells, which subsequently generate a secondary vesicular output capable of influencing regenerative cell populations. This experimental design aimed to dissect the multilayered molecular interactions that couple immune regulation with osteogenic signaling in the oral microenvironment. Defining the mechanisms by which naturally occurring vesicles such as ADEVs shape this bidirectional interface may facilitate the development of next-generation, cell-free therapeutics for oral and maxillofacial regeneration, particularly in clinical scenarios that require targeted immunoregenerative modulation. Moreover, harnessing PDEVs to direct macrophage functional states could offer a valuable adjunct to current grafting approaches and biomaterial-based interventions, thereby expanding the therapeutic toolkit available to regenerative dentistry.

## 2. Results

### 2.1. Physicochemical and Morphological Characterization of Apple-Derived Extracellular Vesicles (ADEVs)

The vesicular preparation obtained from *Malus domestica* underwent an extensive physicochemical evaluation to define the particle size profile and the morphological features at high resolution. The quantification and size distribution of ADEVs were determined using Tunable Resistive Pulse Sensing (TRPS), which measures particles as they pass one at a time through a nanoscale pore embedded in an elastic membrane. When a particle moves through the pore, it causes a temporary increase in electrical resistance across the membrane. These brief resistance changes, known as resistive pulses, correspond to the size of the particle. By counting the number of pulses, the instrument calculates the particle concentration. For the extracted product from *Malus domestica*, TRPS measurements revealed a vesicle population with a mean particle diameter centered around 110 nm, accompanied by a distribution width of approximately ±30 nm. The analysis indicated that the vast majority of vesicles clustered within the 90-180 nm interval, demonstrating a high level of consistency in the isolation workflow and confirming that the preparation predominantly consisted of nanosized structures typical of PDEVs (Figure 2A). Particle concentration plots further supported the robustness of the preparation, showing well-defined peaks and minimal background noise, indicative of low contamination by non-vesicular material.

To complement the quantitative assessment provided by TRPS, a detailed structural examination was carried out by scanning electron microscopy (SEM). SEM imaging offered direct visualization of vesicle surface topology, enabling assessment of shape, membrane continuity, and overall morphological integrity. The representative high-resolution micrograph in Figure 2B shows uniformly spherical nanoparticles with smooth surfaces and well-defined edges, characteristics consistent with intact lipid-bilayer vesicles. The vesicles exhibited diameters spanning below 200 nm, in excellent agreement with the TRPS-derived size distribution. Importantly, SEM inspection did not reveal major aggregates, membrane collapse, or irregular structures, indicating that the isolation and purification procedures preserved vesicle architecture without inducing mechanical deformation or fusion events. The absence of debris or fragmented lipidic material further attests to the purity of the final preparation.

Collectively, the integration of TRPS-based quantitative profiling with ultrastructural visualization by SEM provides a comprehensive validation of the physical properties of ADEVs, confirming their nanoscale size, structural integrity, and morphological uniformity. These characteristics establish a reliable foundation for their subsequent use in biological assays, ensuring that downstream functional experiments are conducted with highly consistent and well-characterized vesicle preparations.

The microRNA cargo encapsulated within apple-derived nanovesicles (ADEVs) was subjected to an in-depth molecular characterization aimed at defining its regulatory potential and biological relevance. High-resolution sequencing and bioinformatic annotation revealed a complex and selectively enriched small RNA repertoire, consistent with an actively sorted vesicular miRNA profile rather than stochastic cellular leakage. Particular attention was directed toward mdm-miR482a-5p, a *Malus domestica* microRNA belonging to a conserved plant miRNA family known to participate in the fine regulation of stress-response and defense-associated signaling networks. In the plant context, members of the miR482 family have been implicated in post-transcriptional control of nucleotide-binding leucine-rich repeat (NLR) genes and other immune-related transcripts, thereby contributing to the modulation of innate defense thresholds and environmental adaptability. The enrichment of mdm-miR482a-5p within ADEVs suggests a selective packaging mechanism and positions this miRNA as a candidate mediator of vesicle-associated bioactivity. Comparative sequence alignment analysis further revealed a remarkable degree of nucleotide similarity—reaching 91.27% homology—between mdm-miR482a-5p and the precursor sequence of human hsa-miR-21, one of the most extensively characterized regulatory microRNAs in mammalian systems (Table 1). The hsa-miR-21 precursor gives rise to mature miR-21, a central node in the orchestration of inflammatory signaling, macrophage polarization dynamics, and broader immune homeostatic processes. MiR-21 is known to modulate multiple downstream targets involved in NF-κB pathway regulation, cytokine production, apoptosis resistance, and immune cell plasticity, thereby acting as a threshold regulator of inflammatory amplitude rather than a simple binary switch. The high degree of sequence conservation observed between the plant-derived mdm-miR482a-5p and the human miR-21 precursor raises the intriguing possibility of structural and functional convergence across kingdoms, potentially enabling partial molecular mimicry or interaction with conserved post-transcriptional regulatory circuits. Although direct cross-kingdom target engagement requires experimental validation, the observed homology provides a biologically plausible framework for interpreting the immunomodulatory activity of ADEVs in human systems. In this context, plant-derived vesicular miRNAs may contribute to immune recalibration by interfacing with evolutionarily conserved regulatory motifs, thereby offering a mechanistic basis for the attenuation of inflammatory tone observed following ADEV exposure.

### 2.2. Modulation of Macrophage Polarization by ADEVs

The high-resolution scanning electron micrograph in Figure 3A shows human macrophages in intimate contact with ADEVs, which appear distributed across the cell surface as discrete nanoscale structures prior to internalization. This ultrastructural visualization provides the first line of evidence that ADEVs effectively engage the macrophage membrane, establishing the physical interface required for subsequent uptake and intracellular signaling. The juxtaposition of vesicles along membrane ruffles and endocytic invaginations suggests that ADEVs exploit canonical endocytic routes, thereby positioning themselves to influence macrophage immunobiology at the earliest steps of vesicle-cell communication.

To further interrogate this process, ADEVs were fluorescently tagged with PKH26 and tracked in living cells. As shown in Figure 3B, confocal fluorescence microscopy revealed intense and punctate red signals distributed throughout the macrophage cytoplasm, unequivocally confirming vesicle internalization and intracellular trafficking. The spatial pattern of fluorescence, clustered in perinuclear regions and aligned along endosomal compartments, strongly supports the hypothesis that ADEVs reach regulatory hubs of innate immunity, where vesicle cargo may intersect with transcriptional and metabolic programs that dictate macrophage phenotype.

Moreover, quantitative gene-expression analysis demonstrated a striking and coordinated shift in macrophage polarization markers (Figure 3C). Specifically, the expression of inducible nitric oxide synthase (iNOS), a hallmark effector of classically activated, pro-inflammatory M1 macrophages, was significantly downregulated following ADEV exposure. In parallel, transcription of arginase-1 (ARG1), a canonical indicator of M2 polarization associated with tissue repair, extracellular matrix remodeling, and pro-resolving immune states, was markedly elevated. This reciprocal regulation of iNOS and ARG1 constitutes a definitive molecular signature of macrophage reorientation toward an M2-like phenotype. The biological implications of this shift are substantial: M1 macrophages typically sustain inflammatory microenvironments that impair osteogenic differentiation, whereas M2 macrophages secrete trophic factors and anti-inflammatory mediators that promote osteoprogenitor activity, matrix deposition, and regenerative healing. Thus, the ADEV-induced transcriptional program observed here extends far beyond simple polarization markers; it represents a mechanistic inflection point in which macrophages are pushed from an inflammatory, osteoinhibitory profile toward a pro-regenerative and osteopromotive state.

Collectively, the SEM-based structural visualization, the intracellular trafficking confirmed by PKH26 fluorescence, and the transcriptional remodeling highlighted by iNOS suppression and ARG1 induction provide a multifaceted demonstration that ADEVs are potent modulators of macrophage fate. By orchestrating a shift toward a reparative M2 phenotype, ADEVs establish the immunological milieu necessary to facilitate downstream osteogenic commitment in mesenchymal stem cells, thereby offering a compelling mechanistic framework through which ADEVs may indirectly potentiate osteoregeneration.

In addition to qualitative observations, the uptake of ADEVs by macrophages was evaluated quantitatively by enumerating the number of extracellular vesicles internalized per cell. Individual macrophages were analyzed over time, and the number of ADEV particles detected within each cell was recorded to generate a kinetic profile of vesicle uptake (Figure 4). The distribution profile demonstrates that ADEVs internalization is not uniform across the macrophage population. Rather, uptake exhibits a heterogeneous pattern, with the majority of cells internalizing a moderate number of vesicles and a smaller subset displaying higher vesicle accumulation. As illustrated in the graph, the frequency of macrophages increases progressively with the number of internalized vesicles, reaching a peak in the mid-range of vesicle counts, and subsequently declining at higher uptake values. This unimodal distribution indicates that ADEVs internalization follows a graded uptake pattern rather than a binary responder/non-responder model. Importantly, the presence of a well-defined peak suggests that ADEVs uptake is a biologically regulated process rather than a random or stochastic event. The decline in frequency at higher vesicle numbers further indicates that only a limited fraction of macrophages exhibits high-capacity internalization, potentially reflecting intrinsic variability in endocytic activity or activation state within the population. Collectively, these quantitative data confirm that ADEVs are effectively internalized by macrophages and demonstrate measurable vesicle accumulation at the single-cell level. The observed heterogeneity in uptake may contribute to the graded immunomodulatory effects detected in downstream analyses and is consistent with the modest but reproducible polarization shift observed following ADEVs exposure.

To determine whether ADEV treatment alters the regulatory state of inflamed monocytes at the level of post-transcriptional control, we quantified a targeted panel of miRNAs classically associated with inflammatory polarization and resolution, together with central nodes of the PI3K–AKT–mTOR signaling axis. Comparison of inflamed monocytes (CTRL) versus inflamed monocytes treated with ADEV (CTRL+ADEVs) revealed a coherent reconfiguration of miRNA abundance, characterized by the concerted down-modulation of an M1-associated miRNA module and the relative enrichment of a distinct miRNA set linked to anti-inflammatory macrophage biology (Figure 5, left “M1” block vs. right “M2” block). Error bars indicate the dispersion across replicates (as displayed), supporting that the differences represent consistent directional shifts across the measured panel rather than isolated outliers (Figure 5). Within the M1-associated block, ADEV treatment was accompanied by a marked decrease in miR-21 and miR-155-5p, two miRNAs frequently positioned at the interface of inflammatory amplification, macrophage activation state, and feedback control. In the CTRL condition, both miR-21 and miR-155-5p were among the more abundant species in the panel, whereas in CTRL+ADEVs they were uniformly reduced, indicating that ADEVs exposure is associated with dampening of a canonical inflammatory miRNA backbone. A similar ADEV-associated reduction extended to additional miRNAs in this cluster—miR-204-5p, miR-451, miR-125b-5p, miR-181a-5p, miR-193b-3p, and miR-125a-5p—collectively suggesting that ADEVs do not simply affect a single inflammatory miRNA but rather induce a broader reprogramming of the miRNA milieu that typically accompanies inflammatory monocyte/macrophage states. Strikingly, the ADEVs-associated miRNA shift also involved multiple components functionally mapped to the PI3K–AKT–mTOR axis, measured here as Akt2 and key regulatory nodes. In CTRL+ADEV, Akt2 abundance was reduced relative to CTRL, paralleling a general trend across this pathway-related module. Similarly, p110δ (the catalytic subunit commonly associated with leukocyte PI3K signaling), TSC1, TSC2, and p85α showed lower values in CTRL+ADEVs than in CTRL, consistent with coordinated pathway-level remodeling rather than stochastic fluctuations. Notably, PTEN also trended lower in CTRL+ADEV in your dataset. Because PTEN and AKT nodes participate in a tightly coupled feedback architecture, simultaneous modulation of multiple elements is compatible with a network rebalancing rather than a unidirectional “on/off” interpretation of a single gene. Importantly, these measurements—taken as a set—indicate that ADEV exposure is associated with a system-wide shift in the regulatory landscape governing inflammatory signaling amplitude and metabolic/integrative control, as reflected by the combined behavior of Akt2/p110δ/PTEN/TSC1/TSC2/p85α. In contrast to the broad downshift observed across the M1-associated panel, the miRNA set grouped under the M2 side of the axis displayed the opposite directionality: CTRL+ADEV showed a clear relative enrichment of miR-124 and miR-130, accompanied by increases in miR-483, miR-877, miR-337, and miR-546. In the CTRL condition these miRNAs were lower, whereas upon ADEV exposure they increased, yielding an internally consistent signature in which the “M2-associated” miRNA block shifts upward as the “M1-associated” block shifts downward. This coordinated bidirectional remodeling is a defining feature of a state transition at the level of miRNA programs and is difficult to reconcile with a simple global scaling effect (e.g., uniform changes across all miRNAs). Instead, the pattern supports selective remodeling of post-transcriptional regulators consistent with attenuation of inflammatory miRNA drivers together with relative promotion of miRNAs linked to regulatory/pro-resolving phenotypes. Taken together, the dataset indicates that ADEV treatment of inflamed monocytes is associated with a directional miRNA-program shift: (i) a reproducible reduction across an inflammatory/M1-leaning miRNA module (including miR-21 and miR-155-5p), (ii) concomitant remodeling of PI3K–AKT–mTOR–associated nodes (Akt2, p110d, PTEN, TSC1/2, p85a), and (iii) relative enrichment of an M2/anti-inflammatory miRNA block (miR-124, miR-130, miR-483, miR-877, miR-337, miR-546). While these results do not by themselves establish causal mechanisms—particularly given the cross-kingdom nature of the initiating material and the modest magnitude of polarization—the internal coherence of the miRNA panel supports the interpretation that ADEV exposure rebalances the inflammatory set-point of activated monocytes toward a more anti-inflammatory, pro-resolving regulatory configuration, as read out by miRNA expression.

### 2.3. Physicochemical and Morphological Characterization of Macrophage-Derived Extracellular Vesicles After Exposure to ADEVs (MDEVs/ADEVs)

The conditioned medium derived from human macrophages polarized in response to ADEV exposure were harvested to isolate EVs. Then, macrophage-derived EVs (MDEVs/ADEVs) were subjected to a rigorous, multi-modal analytical workflow to define their biophysical properties, molecular identity, and biological competence. Tunable Resistive Pulse Sensing (TRPS) analysis demonstrated that the vesicles fell within the canonical size range expected for small EVs and displayed a unimodal distribution with minimal polydispersity (Figure 6A). These metrics confirmed that macrophage vesiculogenesis remained highly controlled despite the immunomodulatory stimulus imparted by ADEVs, and that the isolation procedure yielded a preparation enriched in nanoscale particles consistent with exosomal dimensions.

To complement the hydrodynamic profiling achieved through TRPS measurements, we conducted a detailed ultrastructural evaluation using high-resolution SEM (Figure 6B). The microscope observations revealed a population of discrete, spherical vesicles with sharply contoured membranes and uniform morphology, reaffirming the nanoscale dimensions observed by TRPS and providing direct visual confirmation of vesicle integrity. The absence of membrane collapse, aggregation artifacts, or contaminating debris further attests to the quality and purity of the isolated EVs, indicating that their structural fidelity had been preserved throughout the isolation and purification process.

To definitively authenticate the vesicular nature of the isolated particles, we employed a targeted profiling platform designed to detect hallmark protein signatures associated with extracellular vesicles (Figure 6C). A slight positivity for GM130 was detected; however, this finding is considered not biologically relevant in light of the strong and consistent positivity observed for established extracellular vesicle–associated markers. Taken together, these results support the enrichment of ADEVs, while the minimal GM130 signal is likely attributable to technical background rather than significant Golgi-derived contamination. This analysis demonstrated robust expression of classical EV-associated markers, thereby validating the molecular identity of the preparation and excluding the possibility of co-isolated non-vesicular contaminants. Together, the TRPS, SEM, and semi-quantitative EVs marker array establish a coherent and internally consistent profile of ADEV-conditioned macrophage EVs (MDEVs/ADEVs) as structurally intact, size-homogeneous, and molecularly bona fide vesicles.

We next interrogated the bioactive cargo of these vesicles, focusing on microRNAs known to regulate inflammatory resolution and tissue repair. Quantitative analysis revealed a pronounced enrichment of miR-146a and miR-125, two central orchestrators of macrophage immunoregulatory responses and modulators of downstream stromal cell behavior (Figure 6D). The dominance of these microRNAs within the EV cargo suggests that ADEV-induced macrophage polarization does not merely alter cellular phenotype but also imprints a reparative molecular signature onto the vesicular output, endowing the secreted EVs with the capacity to influence recipient cell populations in a manner aligned with regenerative processes.

### 2.4. Modulation of Osteoregenerative Properties in Dental Pulp Stem Cells (DPSCs)

To investigate whether these vesicles can transmit macrophage-derived regulatory cues to osteoprogenitor cells, we administered the MDEVs/ADEVs to DPSCs. Confocal microscopy demonstrated robust vesicle uptake, with fluorescently labeled MDEVs/ADEVs accumulating throughout the cytoplasm and localizing within vesicular and perinuclear compartments, indicative of active endocytic processing (Figure 6E). The efficient internalization of MDEVs/ADEVs by DPSCs reveals a functional intercellular communication axis through which ADEV-mediated immunomodulation can be relayed to mesenchymal populations central to osteoregeneration. Collectively, these findings establish that ADEV-conditioned macrophages release a vesicular secretome that is structurally coherent, molecularly enriched in immunoregulatory microRNAs, and readily internalized by DPSCs. This multi-layered evidence supports a model in which PDEVs indirectly shape osteogenic trajectories by reprogramming macrophage vesicle output, thereby enabling an immune-to-stromal signaling cascade that modulates the regenerative potential of dental pulp stem cells.

To confirm the effective internalization of MDEVs/ADEVs by DPSCs, vesicle uptake was quantitatively assessed. Individual DPSCs were analyzed, and the number of internalized vesicles per cell was enumerated to generate a frequency distribution of vesicle uptake (Figure 7). The resulting distribution indicates robust MDEVs/ADEVs internalization across the DPSC population. Similar to macrophages, uptake was not uniform; instead, it followed a heterogeneous, unimodal distribution. The frequency of DPSCs increased progressively with vesicle number, reaching a clear maximum in the intermediate-to-high uptake range before declining at higher counts. This pattern suggests that MDEVs/ADEVs uptake by DPSCs is a regulated and graded process rather than a stochastic event confined to a minor subpopulation. Notably, the peak frequency observed at higher vesicle counts compared to macrophages suggests that DPSCs may exhibit substantial vesicle internalization capacity. The distribution tail extending toward higher uptake values indicates the presence of a subset of highly responsive cells with elevated endocytic activity. Conversely, the lower-frequency extremes at minimal uptake levels demonstrate that nearly all DPSCs internalized at least some MDEVs/ADEVs, supporting the notion of broad cellular accessibility. The quantitative uptake profile in DPSCs is consistent with the observed transcriptional remodeling toward osteogenic and angiogenic programs. Efficient vesicle internalization provides a mechanistic basis for direct MDEVs/ADEV-mediated modulation of DPSC signaling pathways. The heterogeneous yet widespread uptake pattern may also explain the graded nature of the downstream gene expression responses observed in the regenerative signature. Collectively, these data confirm that MDEVs/ADEVs are efficiently internalized by DPSCs and establish a quantitative framework linking vesicle uptake to subsequent cellular reprogramming.

To further investigate whether polarization of ADEV-derived macrophages influences osteoregenerative processes, we exposed DPSCs to EVs derived from ADEV-preconditioned macrophages or from untreated macrophages. To assess the osteoinductive potential of the EVs, we analyzed the expression of key osteogenic genes using quantitative real-time PCR (RT-qPCR) and the alkaline phosphatase (ALP) enzymatic activity at multiple time points. Figure 8 reports the results related to the following conditions: (i) standard osteogenic medium (DPSC-OS), (ii) exposure to EVs from untreated THP-1 (MDEVs), and (iii) exposure to EVs from ADEV-treated THP-1 (MDEVs/ADEVs). At day 3, the osteogenic markers BMP2, RUNX2, osteopontin (OSP), and osteocalcin (OSC) were expressed at basal levels in all experimental groups. However, DPSCs treated with MDEVs/ADEVs already exhibited a modest upregulation of BMP2 and RUNX2, suggesting early activation of the osteogenic program. At day 14, a significant increase in RUNX2 and BMP2 expression was observed in the MDEVs/ADEVs group compared to both DPSC-OS and MDEVs groups, indicating enhanced commitment toward osteoblast lineage. Notably, OSP and OSC, typically associated with matrix maturation and late-stage mineralization, showed marked upregulation only at day 21 in the MDEVs/ADEVs group. This progressive and coordinated expression pattern suggests that EVs derived from ADEV-treated macrophages not only trigger early osteogenic signaling but also sustain differentiation over time. In contrast, MDEVs induced only a mild and delayed response, underscoring the specific regenerative potential conferred by the immunomodulatory conditioning. ALP activity was measured at days 3, 7, and 21 to assess functional osteogenic activation. At day 3, activity levels were comparable among all groups, confirming a similar baseline state. However, by day 7, DPSCs treated with MDEVs/ADEVs exhibited a significant increase in ALP activity, surpassing both control and MDEV conditions. This enhancement was maintained and further amplified by day 21, indicating sustained osteogenic differentiation. These results confirm that the EVs derived from an anti-inflammatory macrophage phenotype are functionally capable of stimulating osteogenic activity in DPSCs.

To further characterize the molecular signature induced by MDEVs/ADEVs in DPSCs, we performed RT-qPCR-based profiling of a panel of genes involved in inflammation, angiogenesis, and extracellular matrix (ECM) remodeling after treatment with MDEVs/ADEVs (Table 2).

Transcriptomic profiling of treated DPSCs revealed a coordinated remodeling of gene expression consistent with a transition from an inflammatory microenvironment toward a pro-regenerative osteogenic niche. The transcriptional architecture observed was not limited to isolated marker changes but instead reflected activation of integrated regulatory modules governing inflammation resolution, osteoblast lineage commitment, extracellular matrix synthesis, mineralization, and vascular coupling.

Among the most significantly downregulated transcripts were IL-1α, IL-1β, TNF-α, and IL-6, indicating robust suppression of canonical NF-κB–driven inflammatory signaling. This reduction is mechanistically relevant, as sustained IL-1β and TNF-α signaling are well-established inhibitors of osteoblast differentiation through repression of RUNX2 and disruption of BMP/Wnt pathways. Concomitant reduction in CXCL8 further suggests attenuation of neutrophil-driven amplification loops that perpetuate tissue damage. Collectively, this inflammatory suppression likely establishes a permissive environment for lineage specification and matrix deposition. The downregulation of MMP9 additionally indicates a shift away from matrix degradation and inflammatory remodeling toward constructive extracellular matrix assembly, consistent with a transition from early injury response to repair-phase dynamics.

In parallel with inflammatory suppression, we observed robust upregulation of the core osteogenic transcriptional axis. RUNX2, the master regulator of osteoblast lineage commitment, was significantly induced, accompanied by increased expression of its downstream effector SP7 (Osterix), which is required for terminal osteoblast differentiation. Upregulation of DLX5 and MSX2 further supports activation of upstream developmental programs that reinforce RUNX2 transcriptional activity. This transcriptional cascade was accompanied by activation of canonical osteoinductive signaling pathways, including BMP2/BMP4 and their downstream effector SMAD1, as well as components of the Wnt/β-catenin axis (WNT3A, CTNNB1). These pathways converge to stabilize osteogenic gene expression, promote progenitor commitment, and drive matrix-producing phenotypes. The structural and functional maturation of osteoblasts was supported by increased expression of COL1A1 and COL1A2, encoding the principal fibrillar collagens of bone matrix. Elevated ALPL expression indicates early mineralization competence, while induction of BGLAP (osteocalcin) reflects late-stage matrix maturation and mineral deposition. Upregulation of SPP1 (osteopontin) suggests enhanced matrix organization and cell–matrix interaction, facilitating osteoblast anchoring and mineral nucleation. Together, these markers delineate a transcriptional progression from lineage commitment through extracellular matrix synthesis to mineralization readiness. Bone regeneration is inseparable from vascular remodeling. The treated DPSCs displayed a coordinated induction of angiogenic mediators, including VEGFA and its receptor KDR, as well as ANGPT1, ANGPT2, and PDGFB. These factors orchestrate endothelial activation, vessel sprouting, pericyte recruitment, and vessel stabilization. Upregulation of HIF1A further suggests adaptation to localized hypoxic cues, a known trigger for osteo-angiogenic coupling. The induction of NOS3 implies enhanced endothelial nitric oxide production, supporting vascular tone and perfusion within the regenerating niche. This vascular gene signature indicates the establishment of a microenvironment capable of sustaining nutrient delivery, progenitor recruitment, and matrix maturation.

To evaluate osteogenic differentiation, mineralized matrix formation was assessed using Alizarin Red S staining, a well-established assay for detecting calcium deposition during osteoblast maturation. Following induction of differentiation, cultures were stained and mineral deposition was quantified.

**Figure 9:**
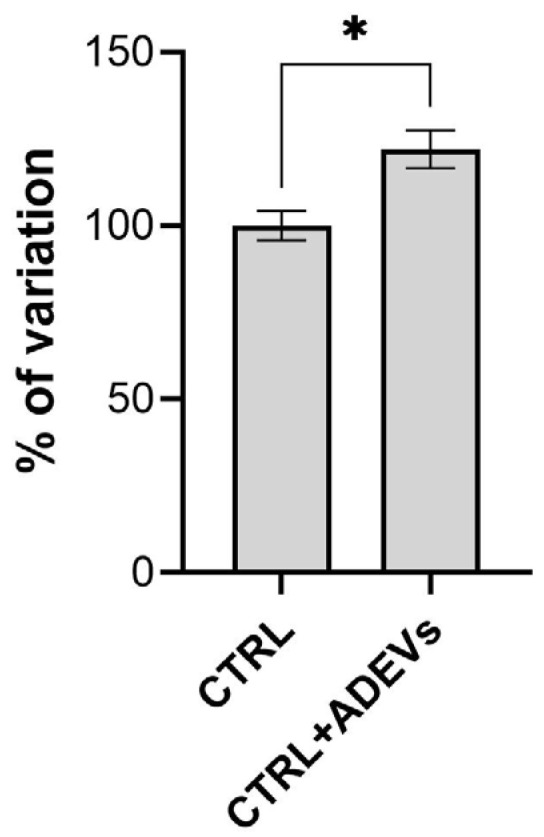
Mineralized matrix quantification by Alizarin Red S staining. Calcium deposition was assessed in differentiated DPSCs treated with MDEVs and with MDEVs/ADEVs. Asterisks indicate statistically significant differences between groups (* *p* < 0.05).

**Figure 1:**
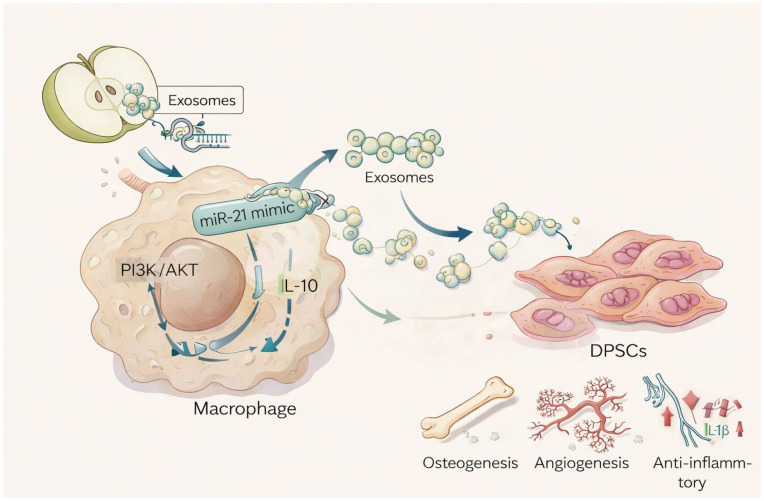
Representative image showing ADEVs internalized by macrophages and subsequent release of macrophage-derived EVs that can indirectly influence target cells, such as DPSCs, promoting beneficial effects.

**Figure 2:**
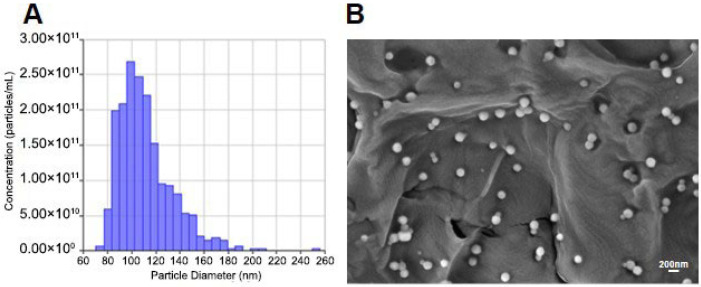
Apple-derived Extracellular Vesicles (ADEVs). (**A**) Quantification (particles/mL) and size distribution (nm) of ADEVs via Tunable Resistive Pulse Sensing. (**B**) Representative image of ADEVs by Scanning Electron Microscopy (SEM). The matrix-like structures visible in the background correspond to the support matrix used for electron microscopy sample preparation. These structures are not part of the purified ADEV fraction but derive from the grid/support material employed during imaging. Scale bar: 200 nm.

**Figure 3:**
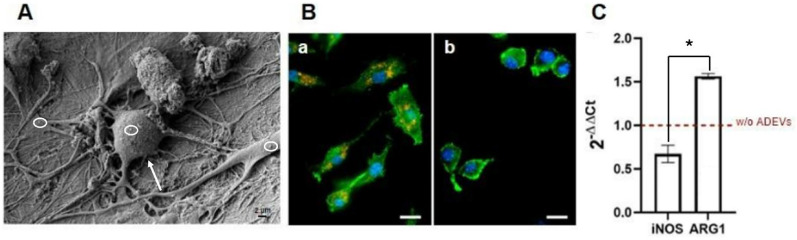
Modulation of Macrophage Polarization by apple-derived extracellular vesicles (ADEVs). (**A**) Representative high-resolution SEM image of human macrophages treated with ADEVs. Macrophages are indicated by arrow, while ADEVs are highlighted by circles. Scale bar: 2 µm. (**B**) Confocal fluorescence microscopy of human macrophages treated with (**B**-**a**) red PKH26-labeled ADEVs and (**B**-**b**) labeled phosphate-buffered saline (PBS) (control). Actin filaments were stained with green phalloidin, and nuclei were counterstained with Hoechst 33342 (blue). Scale bar: 25 µm. (**C**) Gene expression analysis of the pro-inflammatory M1 marker inducible nitric oxide synthase (iNOS) and the M2 polarization marker arginase-1 (ARG1). Asterisks indicate statistically significant differences between groups (* *p* < 0.05).

**Figure 4:**
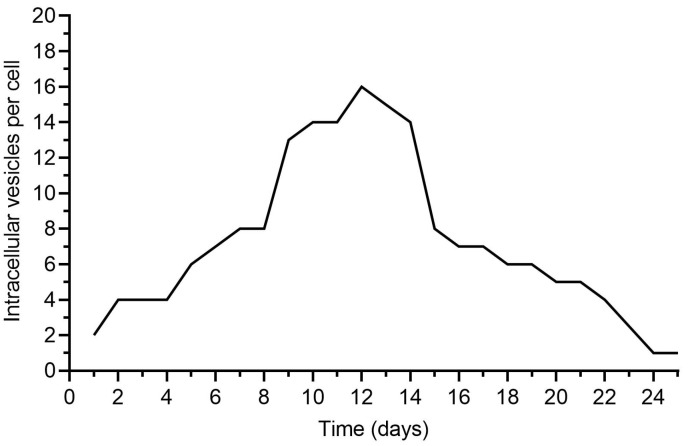
Time-course of ADEV internalization in macrophages. Quantitative assessment of vesicle uptake was monitored over a 25-day period. The graph illustrates the kinetics of intracellular ADEV accumulation, characterized by a progressive increase that peaks at day 12, followed by a gradual decline.

**Figure 5:**
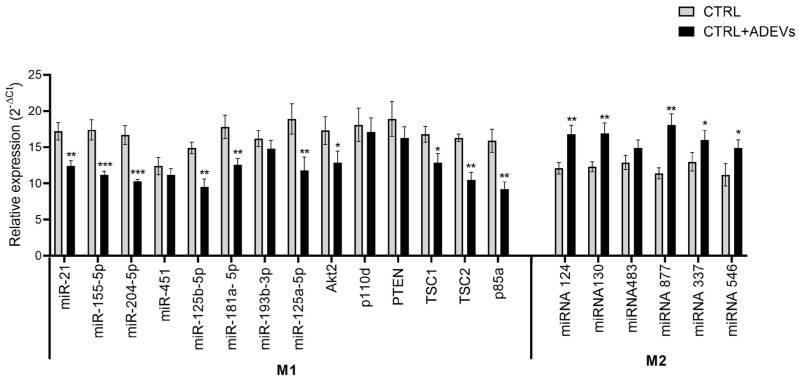
ADEV-mediated modulation of miRNA expression. Relative expression levels were quantified in inflamed monocytes (CTRL) versus those treated with ADEVs (CTRL+ADEVs). Statistical comparison was performed between the two groups; asterisks indicate significance (* *p* < 0.05, ** *p* < 0.01, *** *p* < 0.001).

**Figure 6:**
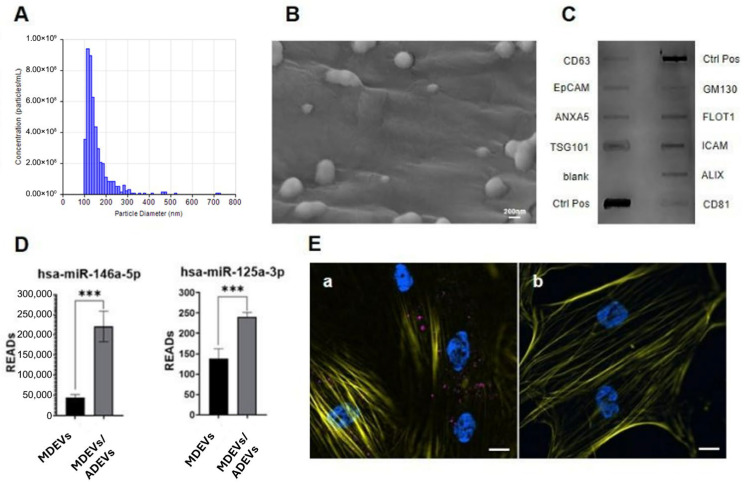
Characterization of macrophage-derived extracellular vesicles after exposure to ADEVs (MDEVs/ADEVs). (**A**) Particle size distribution (nm) of MDEVs/ADEVs via TRPS Analysis. (**B**) Representative high-resolution SEM image of MDEVs/ADEVs. Scale bar: 200 nm. (**C**) Semi-quantitative antibody array of common EVs markers, including CD63, CD81, ALIX, and TSG101 alongside controls for cellular contamination (GM130) (**D**) microRNA cargo of MDEVs: miR-146a and miR-125 reads in MDEVs derived from treated (MDEVs/ADEVs) and not-treated (MDEVs) macrophages. Asterisks indicate statistically significant differences between groups (*** *p* < 0.001). (**E**) Confocal fluorescence microscopy of dental pulp stem cells (DPSCs) treated with (**E**-**a**) red PKH26-labeled MDEVs/ADEVs and (**E**-**b**) labeled PBS (control). Actin filaments were stained with green phalloidin, and nuclei were counterstained with Hoechst 33342 (blue). Scale bar: 25 µm.

**Figure 7:**
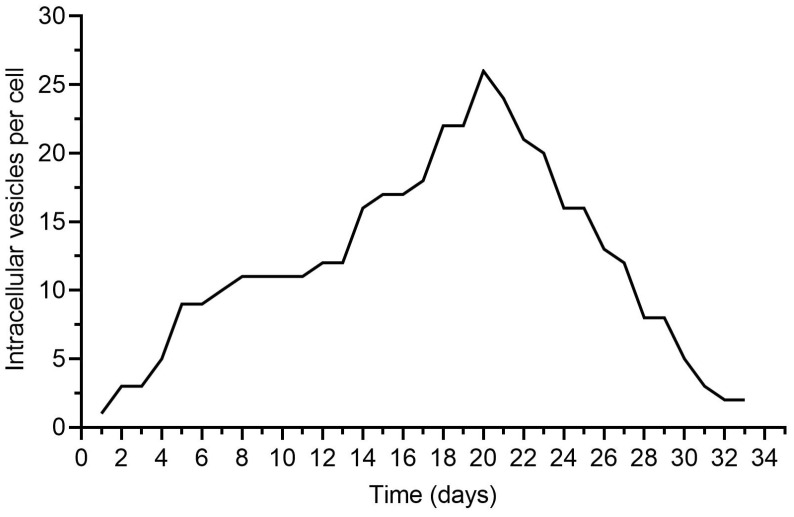
Time-course of MDEVs/ADEVs internalization in Dental Pulp Stem Cells (DPSCs). Quantitative analysis of vesicle uptake in DPSCs was monitored over a 34-day period of time. The graph illustrates the kinetics of intracellular MDEVs/ADEVs accumulation, showing a progressive increase that peaks at day 20, followed by a gradual decline in vesicle content.

**Figure 8:**
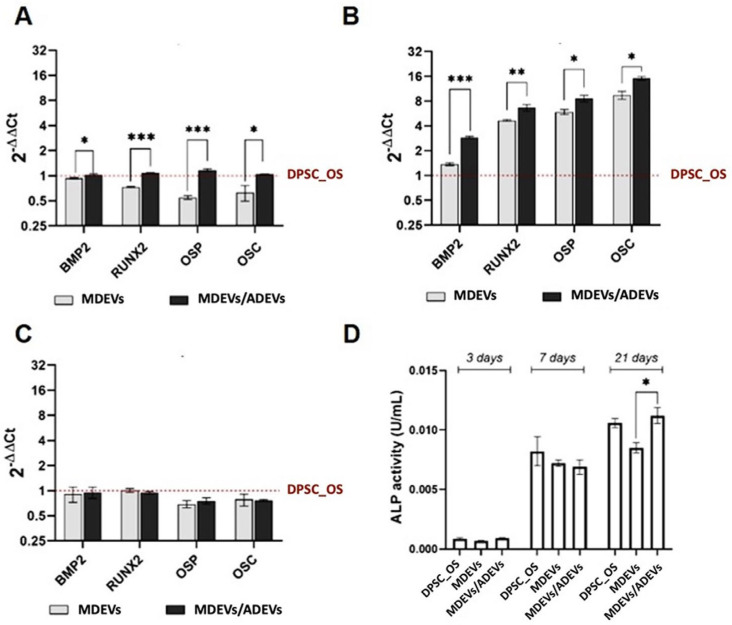
Gene Expression of osteogenic markers and alkaline phosphatase (ALP) enzymatic activity in dental pulp stem cells (DPSCs). Gene expression of osteogenic differentiation markers BMP2, RUNX2, OSP, and OSC after (**A**) 3, (**B**) 14, and (**C**) 21 days of treatment with EVs from ADEV-treated THP-1 (MDEVs/ADEVs), EVs from untreated THP-1 (MDEVs), and standard osteogenic medium (DPSC-OS). (**D**) ALP activity at each treatment point. Asterisks indicate statistically significant differences between groups (One-way ANOVA, * *p* < 0.05, ** *p* < 0.01, *** *p* < 0.001).

As shown in the graph (Figure 9), a clear increase in Alizarin Red staining was observed in the MDEVs as a control versus MDEVs/ADEVs. Quantitative analysis demonstrated a marked elevation in mineralized matrix deposition, indicating enhanced osteogenic differentiation in the treated group. The increase in calcium accumulation reflects functional progression toward osteoblastic maturation and extracellular matrix mineralization.

**Table 2:** Gene expression profile in dental pulp stem cells treated with MDEVs/ADEVs. Log fold change expression of key inflammatory, angiogenic, and extracellular matrix remodeling genes in ADEV-treated DPSCs compared to untreated controls. A positive (+) value indicates upregulation, while a negative (−) value indicates downregulation of gene expression.

Symbol	Entrez Gene Name	Ensemble	Expr Log Ratio	Biological Interpretation
IL-1α	interleukin 1 alpha	ENSG00000115008	−3.850	Strong suppression of early inflammatory cascade
IL-1β	interleukin 1 beta	ENSG00000125538	−3.420	Reduced osteoclast activation signaling
TNF alfa	tumor necrosis factor alpha	ENSG00000232810	−3.980	Decreased NF-κB driven inflammatory inhibition of osteogenesis
IL-6	interleukin 6	ENSG00000136244	−3.765	Reduced chronic inflammatory signaling
CXCL8	C-X-C motif chemokine ligand 8	ENSG00000169429	−3.210	Lower neutrophil recruitment and inflammatory amplification
MMP9	matrix metallopeptidase 9	ENSG00000100985	−2.345	Reduced matrix degradation phase
RUNX2	runt related transcription factor 2	ENSG00000124813	+3.875	Master regulator of osteoblast lineage commitment
SP7	osterix	ENSG00000107518	+4.102	Terminal osteoblast differentiation driver
ALPL	alkaline phosphatase	ENSG00000162551	+3.462	Early mineralization marker
COL1A1	collagen type I alpha 1	ENSG00000108821	+3.995	Major structural bone matrix protein
COL1A2	collagen type I alpha 2	ENSG00000164692	+3.624	Matrix fibril stabilization
BGLAP	osteocalcin	ENSG00000106812	+4.580	Late-stage mineralization marker
SPP1	osteopontin	ENSG00000118785	+3.217	Matrix organization and osteoblast adhesion
BMP2	bone morphogenetic protein 2	ENSG00000125378	+2.944	Induces osteogenic differentiation cascade
BMP4	bone morphogenetic protein 4	ENSG00000125386	+2.508	Synergizes with BMP2 in bone formation
VEGFA	vascular endothelial growth factor A	ENSG00000112715	+3.106	Drives angiogenesis coupled to osteogenesis
ANGPT1	angiopoietin 1	ENSG00000154188	+2.012	Vessel stabilization and maturation
ANGPT2	angiopoietin 2	ENSG00000091879	+2.865	Vascular remodeling during regeneration
PDGFB	platelet derived growth factor B	ENSG00000100345	+2.311	Recruitment of pericytes and vascular support cells
HIF1A	hypoxia inducible factor 1 alpha	ENSG00000100644	+1.842	Hypoxia-adaptive pro-angiogenic signaling
DLX5	distal-less homeobox 5	ENSG00000144355	+3.215	Upstream activator of RUNX2 transcription
MSX2	msh homeobox 2	ENSG00000120149	+2.988	Promotes osteoblast differentiation commitment
WNT3A	Wnt family member 3A	ENSG00000108379	+2.704	Activates canonical Wnt/β-catenin osteogenic signaling
CTNNB1	beta-catenin	ENSG00000168036	+2.412	Central mediator of Wnt-driven osteogenesis
FGF2	fibroblast growth factor 2	ENSG00000138685	+2.563	Enhances proliferation and early osteoprogenitor expansion
IGF1	insulin-like growth factor 1	ENSG00000121410	+2.780	Promotes matrix synthesis and bone growth
NOS3	endothelial nitric oxide synthase	ENSG00000164867	+1.980	Supports angiogenesis and vascular tone
KDR	VEGF receptor 2	ENSG00000128052	+2.346	Endothelial responsiveness to VEGFA
SMAD1	SMAD family member 1	ENSG00000170365	+2.115	BMP pathway signal transduction mediator

Human miRNA Mature Sequences (5′→3′)
**miRNA (Homo sapiens)****Arm****Mature Sequence (RNA, 5′→3′)**miR-215pUAGCUUAUCAGACUGAUGUUGAmiR-1555pUUAAUGCUAAUCGUGAUAGGGGUUmiR-2045pUUCCCUUUGUCAUCCUAUGCCUmiR-451a-AAACCGUUACCAUUACUGAGUUmiR-125b5pUCCCUGAGACCCUAACUUGUGAmiR-181a5pAACAUUCAACGCUGUCGGUGAGUmiR-193b3pAACUGGCCCUCAAAGUCCCGCUmiR-125a5pUCCCUGAGACCCUUUAACCUGUGAmiR-1243pUAAGGCACGCGGUGAAUGCCAAmiR-130a3pCAGUGCAAUGUUAAAAGGGCAUmiR-483-5p5pAAGACGGGAGGAAAGAAGGGAGmiR-483-3p3pUCACUCCUCUCCUCCCGUCUUmiR-877-5p5pGUAGAGGAGAUGGCGCAGGGmiR-877-3p3pUCCUCUUCUCCCUCCUCCCAGmiR-337-3p3pCUCCUAUAUGAUGCCUUUCUUCmiR-337-5p5pGAACGGCUUCAUACAGGAGUU

**Figure 10:**
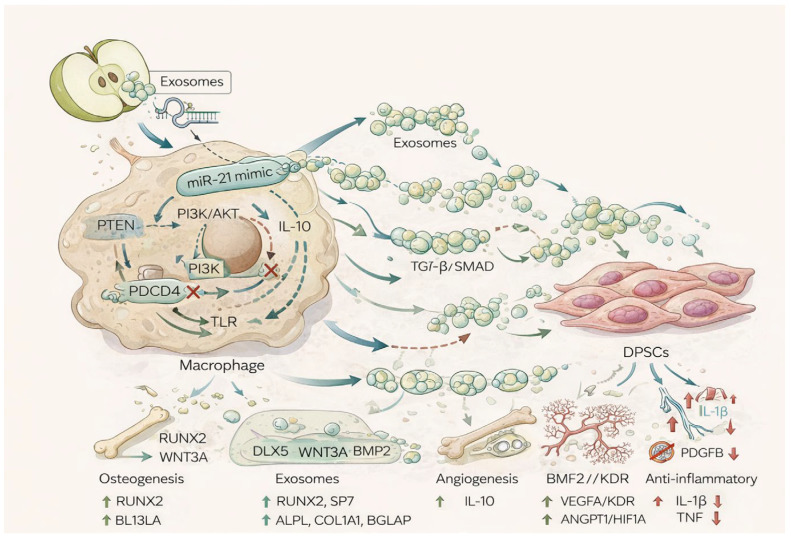
Schematic illustration of the osteoregenerative effects of ADEV-conditioned macrophage EVs on Dental Pulp Stem Cells (DPSCs). Apple-derived extracellular vesicles (ADEVs), enriched in a small RNA exhibiting sequence similarity to hsa-miR-21, induced a reproducible anti-inflammatory shift in macrophages, resulting in attenuation of pro-inflammatory signaling without cytokine amplification. EVs released by these conditioned macrophages promoted coordinated suppression of inflammatory mediators and robust activation of osteogenic (RUNX2/SP7, BMP–SMAD, Wnt/β-catenin) and angiogenic (VEGFA-related) programs in DPSCs, supporting an immune-mediated pro-regenerative transcriptional architecture.

Apple-derived extracellular vesicles (ADEVs), enriched in a small RNA exhibiting sequence similarity to hsa-miR-21, induced a consistent anti-inflammatory shift in macrophages. Although the magnitude of polarization was modest, the directionality of the response was unambiguous and reproducible, indicating attenuation rather than activation of inflammatory pathways. This effect was not associated with heightened pro-inflammatory cytokine production, but instead reflected a reduction in inflammatory tone, consistent with a pro-resolving macrophage phenotype. Given the well-established role of macrophages as regulators of mesenchymal stem cell behavior through exosome-mediated intercellular communication, we investigated whether this anti-inflammatory macrophage state was associated with downstream transcriptional effects in DPSCs. DPSCs exposed to macrophage-conditioned signals displayed a coordinated gene expression program characterized by marked suppression of inflammatory mediators, including IL-1α, IL-1β, TNF-α, and IL-6. These cytokines are known inhibitors of osteogenic differentiation through interference with RUNX2 activity and BMP/Wnt signaling; their downregulation therefore represents a permissive condition for bone formation. The anti-inflammatory environment was further supported by the absence of inflammatory amplification markers in DPSCs, indicating that macrophage-derived signals did not propagate immune stress to mesenchymal cells. In parallel, we observed robust upregulation of osteogenic master regulators RUNX2 and SP7 (Osterix), along with upstream transcription factors DLX5 and MSX2. Canonical osteoinductive pathways, including Wnt/β-catenin (WNT3A, CTNNB1) and BMP signaling (BMP2, BMP4, SMAD1), were significantly induced. Matrix deposition and mineralization markers such as COL1A1, COL1A2, ALPL, and BGLAP were elevated, indicating progression toward osteoblastic maturation. Concomitantly, angiogenic mediators including VEGFA, KDR, ANGPT1, ANGPT2, PDGFB, NOS3, and HIF1A were upregulated, consistent with activation of osteo-vascular coupling programs (Figure 10). The integration of osteogenic and angiogenic modules in the context of inflammatory suppression reflects a coordinated regenerative transcriptional architecture. Macrophages are known to dynamically modulate their exosomal cargo in response to activation state. An anti-inflammatory polarization can reshape vesicular content toward pro-resolving cytokines, growth factors, and regenerative microRNAs. Although we did not directly profile macrophage-derived exosomes in this study, the combined suppression of inflammatory genes and induction of osteo-angiogenic pathways in DPSCs strongly supports the interpretation that apple-derived EVs initiate an anti-inflammatory macrophage shift, which in turn generates a secondary exosomal or paracrine signal favoring tissue regeneration. Importantly, given the vegetal origin of the initiating vesicles and the modest magnitude of macrophage polarization observed, these findings should be interpreted conservatively. Nevertheless, the consistent alignment between anti-inflammatory macrophage modulation and robust osteogenic and angiogenic transcriptional activation in DPSCs indicates that even subtle immune reprogramming is sufficient to produce biologically meaningful pro-regenerative outcomes.

## 3. Discussion

The establishment of a stable and functional osteointegrative interface remains a fundamental prerequisite for the long-term success of dental implants [38,39,40,41]. Yet, even with major technological progress in implant macro- and micro-design, the predictability of early healing continues to be jeopardized by inflammatory dysregulation, suboptimal osteogenic signaling, and systemic conditions that compromise the host regenerative response. Increasing evidence underscores that the earliest phases of healing, dominated by macrophage activity and osteoimmunological cross-talk, play a decisive role in determining the trajectory of bone regeneration. An orchestrated transition of macrophages from an M1-dominant inflammatory state to an M2 pro-resolutive and pro-regenerative phenotype is essential for MSC recruitment, differentiation, and subsequent deposition of mineralized extracellular matrix [15,19].

Our data support a model in which *Malus domestica*–derived extracellular vesicles (ADEVs) act primarily as upstream immunoregulatory cues rather than as direct osteoinductive instructive agents. First, molecular profiling of ADEV small RNAs identified a plant miRNA (mdm-miR482a-5p) displaying high sequence identity (91.67%) to the human hsa-miR-21 precursor. Given the established role of miR-21 in shaping inflammatory amplitude and macrophage plasticity, this cross-kingdom similarity provides a biologically plausible rationale—without implying direct target equivalence—for why ADEVs may interface with mammalian immune regulation. Importantly, we treat this observation as hypothesis-generating, consistent with a scenario of regulatory convergence rather than definitive functional mimicry, and it sets a mechanistic context for the cellular effects observed downstream.

At the cellular level, human macrophages robustly recognize and internalize ADEVs, as documented by ultrastructural and fluorescence evidence and further supported by quantitative single-cell uptake distributions. Notably, internalization followed a graded, unimodal profile, arguing against a rare-cell phenomenon and instead suggesting regulated entry into biologically relevant compartments. ADEV exposure induced a modest but reproducible shift toward a pro-resolving phenotype, captured by reciprocal regulation of canonical polarization markers (iNOS down, ARG1 up) and corroborated by directional remodeling of a targeted miRNA panel. While the magnitude of polarization is not extreme—and we avoid overstating it—the internal coherence across marker classes supports the interpretation that ADEVs attenuate inflammatory tone rather than activate inflammatory pathways. In our experimental setting, macrophage polarization toward an M2-like phenotype was defined on the basis of established phenotypic and molecular markers, including the upregulation of canonical M2-associated markers and the modulation of inflammatory mediators. IL-4/IL-13 were incorporated as functional reference controls to validate the responsiveness and dynamic range of the polarization model, thereby providing a benchmark for comparison within the same experimental framework. Importantly, the observed phenotypic transition induced by our formulation should be interpreted as functional polarization rather than evidence of shared intracellular signaling pathways with cytokine-driven M2 activation. The present study was designed to characterize the phenotypic outcome of macrophage reprogramming, whereas detailed mechanistic dissection of pathway convergence or divergence will require dedicated future investigations.

Crucially, the most consequential outcome emerged not at the level of macrophage markers per se, but in the functional relay to mesenchymal stromal cells. When DPSCs were exposed to EVs produced by ADEV-conditioned macrophages (MDEVs/ADEVs), they displayed a coordinated transcriptional architecture characterized by suppression of inflammatory mediators (IL-1α, IL-1β, TNF-α, IL-6, CXCL8) alongside robust activation of osteogenic master regulators (RUNX2, SP7, DLX5, MSX2), reinforcement of BMP–SMAD and Wnt/β-catenin signaling (BMP2/4, SMAD1, WNT3A, CTNNB1), induction of matrix/mineralization genes (COL1A1/2, ALPL, BGLAP, SPP1), and parallel engagement of angiogenic/osteo-vascular coupling programs (VEGFA/KDR, ANGPT1/2, PDGFB, HIF1A, NOS3). Functionally, this transcriptional remodeling translated into enhanced osteogenic performance, with greater ALP activity and increased mineral deposition compared with EVs from non-conditioned macrophages. Together, these results indicate that ADEVs can improve osteoregenerative outcomes indirectly, by reprogramming macrophage state and—critically—the bioactivity of the macrophage vesicular secretome that then instructs DPSCs. Within this framework, EVs have emerged as evolutionarily conserved biological vectors capable of reshaping immune and stromal behaviors in situ. While mammalian-derived EVs demonstrate considerable regenerative potential, their translational use is constrained by immunogenicity concerns, limited scalability, and regulatory burdens [27,42,43]. In contrast, PDEVs, particularly those isolated from edible fruits, offer an intrinsically biocompatible, non-immunogenic, and ethically sustainable alternative with increasing relevance for biomedical innovation [31,32,33,44].

Recent evidence has consolidated the concept that plant-derived extracellular vesicles (PDEVs) actively contribute to skeletal homeostasis and bone regeneration through multi-layered regulatory mechanisms. A growing body of literature demonstrates that vesicles isolated from medicinal and edible plants—including Epimedium, Scutellaria baicalensis, sea buckthorn, ginseng, yam, and Gouqi—promote osteoblast differentiation, stimulate angiogenesis, inhibit osteoclastogenesis, and enhance fracture repair in preclinical models [45,46,47,48,49,50,51,52,53]. Mechanistically, these effects have been associated with activation of canonical osteogenic pathways such as PI3K/Akt, BMP–SMAD, Wnt/β-catenin, and VEGF-mediated angiogenesis, as well as with modulation of inflammatory signaling cascades that constrain bone repair. In particular, plant EV-like nanoparticles have been shown to foster osteo-vascular coupling, enhance matrix mineralization, and reshape immune–stromal communication within skeletal tissues, positioning PDEVs as emerging cell-free platforms for bone regenerative strategies [54,55,56,57,58,59,60]. These findings collectively establish that plant EVs are not passive dietary byproducts but biologically active nanostructures capable of orchestrating regenerative programs in bone-related contexts.

Within this expanding landscape, ADEVs derived from *Malus domestica* exhibit distinguishing features that refine and extend current paradigms. While several plant-derived vesicle systems primarily act through direct stimulation of osteoblast lineage commitment or inhibition of osteoclast differentiation, our data indicate that apple-derived EVs operate upstream, recalibrating the inflammatory microenvironment that conditions skeletal repair. This immunoregenerative positioning differentiates ADEVs from previously described osteo-inductive plant vesicles by emphasizing context modulation rather than direct lineage enforcement. Moreover, prior work has demonstrated that apple-derived vesicles possess robust immunomodulatory and microbiota-interacting properties, supporting the concept that their biological activity is intrinsically linked to immune homeostasis. The convergence of immune tuning and osteogenic permissiveness observed in the present study suggests that ADEVs act as systemic regulators of regenerative threshold rather than as isolated osteo-activators. In contrast to engineered or pharmacologically enriched vesicles, ADEVs retain a native molecular complexity and favorable safety profile, offering a scalable, food-derived, biologically integrated platform for skeletal regeneration. Thus, while aligning with the broader evidence that plant EVs support bone repair, our findings uniquely position apple-derived vesicles as modulators of immune resolution that unlock endogenous osteo-vascular programs within inflamed skeletal niches.

In this view, our system, based on the indirect, immune–stromal mechanism, distinguishes our work from a growing literature showing that plant-derived EV-like nanoparticles can promote bone regeneration through more direct effects on osteoblast lineage, angiogenesis, and osteoclast regulation in various models and plant sources. In contrast, we position apple vesicles as context modulators: they recalibrate the inflammatory set-point that determines whether osteogenic and vascular programs can proceed efficiently, a concept particularly relevant in oral sites where inflammatory burden often constrains regeneration. Thus, the novelty of ADEVs lies less in demonstrating yet another pro-osteogenic plant vesicle and more in defining an immunoregenerative cascade in which a food-derived vesicle input reshapes macrophage output EVs, thereby unlocking osteo-angiogenic competence in DPSCs.

From a translational perspective, the findings motivate *in vivo* testing along two complementary lines. First, local delivery paradigms (e.g., defect-adjacent administration or incorporation into compatible biomaterial carriers) could assess whether ADEV conditioning accelerates resolution of early inflammatory responses and improves osteo-vascular integration in clinically relevant settings. Second, mechanistic *in vivo* studies should prioritize dose–response when combined with special supportive matrices to test their in situ release, while using pre-specified endpoints for bone formation and vascularization. In human-facing development, ADEVs offer attractive practical advantages—natural origin, scalability, and favorable tolerability profiles reported for edible plant vesicles—yet cross-kingdom miRNA hypotheses should be advanced with rigor, including validation of cargo persistence, recipient-cell engagement, and downstream pathway modulation in situ. Overall, our data support ADEVs as cell-free immunoregenerative modulators, capable of indirectly enhancing osteogenesis by reprogramming macrophage-derived EV signaling to mesenchymal progenitors.

## 4. Materials and Methods

### 4.1. Apple-Derived Extracellular Vesicles (ADEVs): Isolation and Characterization

Fresh *Malus domestica* var. Golden Delicious apples (Val di Non, Trentino, Italy) were used as the source of ADEVs, as previously described [32]. Briefly, about 500 g of fruit were washed, pulped, and homogenized, and the resulting extract was clarified by sequential centrifugation (650× *g*, 3000× *g*, and 10,000× *g*). The supernatant was filtered through 0.2 µm membranes (GVS, Zola Predosa, Italy) and further purified by centrifugation at 15,000× *g* followed by ultracentrifugation at 110,000× *g* using an Optima L-70 system (Beckman Coulter, Brea, CA, USA). The final vesicle pellet was suspended in phosphate-buffered saline (PBS, Thermo Fisher Scientific, Waltham, MA, USA) and stored at −80 °C until use.

Size distribution and concentration (particles/mL) of ADEVs were measured via Tunable Resistive Pulse Sensing (TRPS) using qNano Instrument (Izon Science, Christchurch, New Zealand), employing an NP100 nanopore (Bio-Rad, Segrate, Italy) stretched to 47 mm [61]. Calibration was performed using CPC100 standard particles (1.7 × 10^13^ particles/mL, average diameter 100 nm) under two pressure settings (10 and 20 mbar). All measurements were executed in triplicate.

### 4.2. Extracellular Vesicles Derived from Human Cells

Human THP-1 monocytes (Resnova, Rome, Italy) were propagated in RPMI 1640 medium supplemented with 2 mM L-glutamine and 10% fetal bovine serum (Euroclone, Pero, Italy), under standard culture conditions (37 °C, 5% CO_2_, humidified atmosphere). Conversion of THP-1 cells into macrophage-like cells was achieved by exposure to Phorbol 12-Myristate 13-Acetate (PMA, 100 ng/mL) for 24 h, after which the cells were allowed to stabilize for an additional 72 h in complete medium.

EVs from THP1 were analyzed by TRPS using qNano Instrument (Izon Science). Particle size distribution and concentration were performed as previously described.

Exosomal protein markers were detected with the membrane-based antibody array (Exo-Check™ Exosome Antibody Array; Systems Biosciences, Palo Alto, CA, USA). The array comprises pre-printed spots for 8 EVs-specific markers (CD63, CD81, ALIX, FLOT-1, ICAM1, EpCAM, ANXA5, and TSG101), cellular contamination (GM130), positive controls and blank control. Images acquired with ChemiDoc System (Bio Rad, Hercules, CA, USA).

### 4.3. Scanning Electron Microscopy (SEM)

To assess the surface morphology of the purified ADEVs and MDEVs/ADEVs, the vesicle suspension was fixed by mixing it 1:1 with 2% glutaraldehyde prepared in phosphate buffer. Following fixation, aliquots were placed onto clean glass coverslips or silicon wafers and allowed to adsorb. The samples were then dehydrated through a graded ethanol series and subjected to critical point drying to preserve the structure. Once dried, the specimens were mounted on aluminum stubs and sputter-coated with a thin conductive layer of gold–palladium to prevent charging during imaging. Morphological evaluation was carried out using a field-emission scanning electron microscope (FE-SEM), which enabled high-resolution visualization of vesicle contours, dimensional uniformity, and membrane ultrastructural features.

### 4.4. Fluorescent Labeling and Confocal Imaging

Human dental pulp mesenchymal stem cells (DPSCs) (Axol Bioscience, Easter Bush, UK) and THP1 cells, purchased from a certified commercial supplier, were cultured according to the manufacturer’s recommended protocols prior to their use in downstream experiments.

For fluorescent staining, cells grown on poly-D-lysine–treated glass coverslips were immobilized using 4% paraformaldehyde. Unreacted aldehydes were neutralized by incubation in 0.1 M glycine prepared in PBS, after which the cells were rendered permeable with 0.1% Triton X-100 and subsequently incubated in PBS containing 2% BSA to minimize nonspecific antibody binding. Actin filaments were labeled using Alexa Fluor 488-phalloidin (Thermo Fisher Scientific), while nuclear DNA was counterstained with Hoechst 33342. 

For visualization of vesicle internalization, ADEVs were fluorescently tagged using PKH26 (Sigma-Aldrich, Merck Group, Darmstadt, Germany). Briefly, 0.8 µL of dye was diluted into 200 µL of Diluent C and combined with the vesicle suspension in PBS to reach a final volume of 400 µL. After a 5-min incubation at ambient temperature, unincorporated dye was removed by centrifugal concentration using 30 kDa Amicon Ultra filters (Millipore, Merck Group, Darmstadt, Germany) at 14,000× *g* for 20 min. The labeled vesicles were then administered to cultured cells following the established treatment schedule. Image acquisition was carried out using a Nikon ECLIPSE Ti confocal (Nikon, Tokio, Japan) platform equipped with a DS-Qi2 camera and 60× oil-immersion objectives (Nikon, Tokio, Japan).

### 4.5. Semi-Quantitative Real-Time PCR

Total RNA was isolated from both stimulated and untreated cultures using the RNeasy Mini Kit (Qiagen, Hilden, Germany), adhering to the supplier’s instructions. The quantity and purity of RNA extracts were evaluated with a NanoDrop™ 2000 spectrophotometer (Thermo Fisher Scientific, Waltham, MA, USA), and only samples exhibiting A260/A280 absorbance ratios within the 1.9–2.1 range were retained for analysis. Residual genomic DNA was removed through on-column DNase treatment to ensure downstream specificity. cDNA was generated from 500 ng of purified RNA using the High-Capacity Reverse Transcription Kit (Applied Biosystems, Thermo Fisher Scientific) in a final reaction volume of 20 µL. The enzymatic synthesis protocol involved an initial primer annealing phase at 25 °C for 10 min, followed by reverse transcriptase activity at 37 °C for 2 h, and final thermal inactivation at 85 °C for 5 min. Quantitative PCR analyses were carried out with PowerUp SYBR Green Master Mix (Thermo Fisher Scientific) on a QuantStudio™ 5 real-time platform (Applied Biosystems, Waltham, MA, USA). Each reaction (20 µL) contained 1 µL of synthesized cDNA, 10 µL of 2× SYBR reagent, and primers at a final concentration of 300 nM. Thermal cycling consisted of a 2-min activation at 95 °C, then 40 amplification cycles comprising denaturation at 95 °C for 15 s, annealing at 60 °C for 30 s, and elongation at 72 °C for 30 s. Melt curve profiling was performed following amplification to verify the generation of a single, specific product. Relative gene expression was normalized against GAPDH or ACTB reference transcripts. 

### 4.6. Small RNA Sequencing and Analyses

Small RNAs were extracted from ADEV samples using an exosome RNA isolation kit according to the manufacturer’s instructions. RNA quantity and quality were assessed using a NanoDrop (Nikon, Tokio, Japan) spectrophotometer and samples were stored at −80 °C until further processing. For miRNA profiling, 250 ng of RNA were used to prepare libraries with the QIAseq miRNA Library Kit (Nikon, Tokio, Japan). Sequencing was performed on an Illumina NovaSeq (San Diego, CA, USA) 6000 platform (2 × 150 bp). miRNAs were identified using QIAseq miRNA-NGS analysis software v.s. 12 (Qiagen) with single-read settings. All experiments were conducted in triplicate. To determine whether a plant-derived microRNA exhibits sequence similarity to hsa-miR-21, we implemented a structured multi-level comparative framework integrating seed-region analysis, pairwise alignment metrics, and short-sequence homology searches. Given the short length of mature microRNAs (typically 20–23 nucleotides), conventional homology thresholds must be interpreted cautiously and contextualized within functional constraints governing microRNA–mRNA target recognition. All analyses were performed using mature microRNA sequences, rather than precursor hairpin transcripts. Human miR-21 (hsa-miR-21-5p) sequence information was retrieved from miRBase and cross-validated against RNAcentral. For each *Malus domestica* microRNA of interest, mature 5p and/or 3p sequences were obtained from curated plant miRNA databases (e.g., miRBase entries specific to *Malus* spp.). Only mature sequences were used for downstream analysis, as target recognition is mediated by the mature strand incorporated into Argonaute complexes. Functional microRNA targeting is predominantly determined by the seed region, defined as nucleotides 2–8 from the 5′ end of the mature miRNA. Consequently, seed-level identity was prioritized as the primary criterion for functional similarity assessment. The seed sequence of hsa-miR-21-5p (nucleotides 2–8) was extracted and compared against the corresponding positions of each plant-derived microRNA. Exact 7-mer seed matches (7/7 identity) were considered indicative of strong functional mimicry potential. Partial matches (6/7 identity) were classified as moderate similarity, whereas lower identity scores were considered unlikely to confer canonical target overlap. Importantly, absence of seed identity precludes classification as “miR-21-like” in a functional sense, regardless of broader sequence resemblance. To quantify overall sequence similarity, mature plant miRNA sequences were aligned against hsa-miR-21-5p using both global (Needleman–Wunsch) and local (Smith–Waterman) alignment algorithms implemented in EMBOSS and equivalent tools. Gap penalties were optimized for short RNA sequences to avoid artificial insertion bias. For each comparison, the following metrics were calculated: percent identity across full-length alignment, number and distribution of mismatches, alignment score, presence or absence of gaps. While overall percent identity provides a descriptive measure of sequence similarity, it is not sufficient to infer functional mimicry in the absence of seed conservation. To complement pairwise analysis, BLASTn searches (Version 6) were conducted using parameters optimized for short sequences (reduced word size, adjusted mismatch penalties). Plant-derived miRNA sequences were queried against a curated human miRNA database to identify the closest human homologs based on sequence identity and alignment score. This approach enabled differentiation between superficial sequence resemblance and biologically meaningful seed conservation.

To determine whether a plant-derived microRNA exhibits sequence similarity to hsa-miR-21, we implemented a structured multi-level comparative framework integrating seed-region analysis, pairwise alignment metrics, and short-sequence homology searches. Given the short length of mature microRNAs (typically 20–23 nucleotides), conventional homology thresholds must be interpreted cautiously and contextualized within functional constraints governing microRNA–mRNA target recognition. All analyses were performed using mature microRNA sequences, rather than precursor hairpin transcripts. Human miR-21 (hsa-miR-21-5p) sequence information was retrieved from miRBase and cross-validated against RNAcentral. For each *Malus domestica* microRNA of interest, mature 5p and/or 3p sequences were obtained from curated plant miRNA databases (e.g., miRBase entries specific to *Malus* spp.). Only mature sequences were used for downstream analysis, as target recognition is mediated by the mature strand incorporated into Argonaute complexes. Functional microRNA targeting is predominantly determined by the seed region, defined as nucleotides 2–8 from the 5′ end of the mature miRNA. Consequently, seed-level identity was prioritized as the primary criterion for functional similarity assessment. The seed sequence of hsa-miR-21-5p (nucleotides 2–8) was extracted and compared against the corresponding positions of each plant-derived microRNA. Exact 7-mer seed matches (7/7 identity) were considered indicative of strong functional mimicry potential. Partial matches (6/7 identity) were classified as moderate similarity, whereas lower identity scores were considered unlikely to confer canonical target overlap. Importantly, the absence of seed identity precludes classification as “miR-21-like” in a functional sense, regardless of broader sequence resemblance. To quantify overall sequence similarity, mature plant miRNA sequences were aligned against hsa-miR-21-5p using both global (Needleman–Wunsch) and local (Smith–Waterman) alignment algorithms implemented in EMBOSS and equivalent tools. Gap penalties were optimized for short RNA sequences to avoid artificial insertion bias. For each comparison, the following metrics were calculated:

- Percent identity across full-length alignment
- Number and distribution of mismatches
- Alignment score
- Presence or absence of gaps

While overall percent identity provides a descriptive measure of sequence similarity, it is not sufficient to infer functional mimicry in the absence of seed conservation. To complement pairwise analysis, BLASTn searches were conducted using parameters optimized for short sequences (reduced word size, adjusted mismatch penalties). Plant-derived miRNA sequences were queried against a curated human miRNA database to identify the closest human homologs based on sequence identity and alignment score. This approach enabled differentiation between superficial sequence resemblance and biologically meaningful seed conservation. A plant-derived microRNA was classified as “miR-21–like” only if: The mature sequence exhibited ≥6/7 seed identity with hsa-miR-21-5p; and Sequences lacking seed conservation were not classified as miR-21 homologs, even if global percent identity exceeded 40–50%. Given the short length of microRNAs, random sequence similarity can occur by chance. Therefore, statistical context is critical when interpreting percent identity values. Moreover, even perfect seed identity does not guarantee functional equivalence, as additional factors—including 3′ supplementary pairing, thermodynamic stability, Argonaute loading compatibility, and intracellular concentration—determine biological activity. Accordingly, sequence similarity analysis should be interpreted as an indicator of potential functional convergence rather than definitive evidence of cross-kingdom targeting.

### 4.7. Statistical Analysis

Statistical evaluation was conducted using GraphPad Prism 8 software version 8.0.0 for Windows (GraphPad Software, San Diego, CA, USA, www.graphpad.com). Differences between two groups were assessed using Student’s *t*-test, whereas comparisons among more than two groups were performed by one-way ANOVA, followed by Bonferroni post hoc multiple comparison test (GraphPad Software). Statistical significance was defined as *p* < 0.05. Confocal microscopy images were analyzed using ImageJ software version 9 to determine the mean vesicle-associated fluorescence intensity per cell. All independent experiments were performed using three biological replicates (n = 3), and results are expressed as mean (SD), unless otherwise specified.

## Figures and Tables

**Table 1:** Percentage of miRNA identity.

mdm-miRNA	hsa-miRNA	Identity
mdm-miR482a-5p	hsa-mir-21 precursor	91.67%

## Data Availability

The original contributions presented in this study are included in the article. Further inquiries can be directed to the corresponding author.

## Abbreviations

ADEVs	Apple-derived Extracellular Vesicles
BMP2	Bone Morphogenetic Protein-2
DPSCs	Dental Pulp Stem Cells
EVs	Extracellular Vesicles
MDEVs	Macrophage-derived Extracellular Vesicles
MDEVs/ADEVs	ADEV-conditioned macrophage EVs
MSCs	Mesenchymal Stem Cells
OSC	Osteocalcin
OSP	Osteopontin
PDEVs	Plant-derived Extracellular Vesicles
RUNX2	RUNX Family Transcription Factor 2
TRPS	Tunable Resistive Pulse Sensing
